# A double-blind randomized study assessing safety and efficacy following one-year adjunctive treatment with bitopertin, a glycine reuptake inhibitor, in Japanese patients with schizophrenia

**DOI:** 10.1186/s12888-016-0778-9

**Published:** 2016-03-15

**Authors:** Yoshio Hirayasu, Shin-Ichi Sato, Hideaki Takahashi, Sayaka Iida, Norifumi Shuto, Seitaro Yoshida, Takashi Funatogawa, Takahito Yamada, Teruhiko Higuchi

**Affiliations:** Department of Psychiatry, Yokohama City University Graduate School of Medicine, Yokohama, Japan; Chugai Pharmaceutical Co., Ltd., Tokyo, Japan; National Center of Neurology and Psychiatry, Tokyo, Japan

**Keywords:** Schizophrenia, Glycine reuptake inhibitor, Bitopertin, NMDA receptor

## Abstract

**Background:**

Bitopertin, a glycine reuptake inhibitor, was investigated as a novel treatment for schizophrenia. We report all the results of a double-blind randomized study assessing safety and efficacy following 52-week adjunctive treatment with bitopertin in Japanese patients with schizophrenia.

**Methods:**

This study enrolled Japanese outpatients with schizophrenia who met criteria for either “negative symptoms”, i.e., patients with persistent, predominant negative symptoms of schizophrenia even after long-term treatment with antipsychotics or “sub-optimally controlled symptoms”, i.e., patients with insufficiently improved symptoms of schizophrenia even after long-term treatment with antipsychotics, respectively. One hundred sixty-one patients were randomly assigned to receive 52-week treatments with bitopertin doses of 5, 10, or 20 mg/day at ratio of 1:5:5, where existing antipsychotics were concomitantly administered. Efficacy endpoints included Positive and Negative Syndrome Scale (PANSS), Clinical Global Impression (CGI), and Personal and Social Performance (PSP). The purpose of the present study is primarily to evaluate the safety, and secondarily to investigate the clinical efficacy of bitopertin.

**Results:**

One hundred fourteen patients (71 %) completed 52-week treatment with bitopertin. Most of the adverse events were mild or moderate in their severity. The patients in the 20-mg group experienced more adverse events than the patients in the other two groups. Common dose-dependent adverse events were somnolence and insomnia associated with worsening schizophrenia. The blood hemoglobin levels gradually decreased from baseline in a dose-dependent manner, but there were no patients with the decrease below 10 g/dL that would have led to their discontinuation. All the efficacy endpoints gradually improved in all the treatment groups for both of the two symptoms, while there were no clear differences among the three dose groups.

**Conclusions:**

Altogether, bitopertin was found to be generally safe and well-tolerated for the treatment of patients with schizophrenia. All three bitopertin treated groups showed improvements in all the efficacy endpoints for both of the two symptoms, i.e., “negative symptoms” and “sub-optimally controlled symptoms”, throughout the duration of the study.

**Trial registration:**

Japan Pharmaceutical Information Center, number JapicCTI-111627 (registered on September 20, 2011)

**Electronic supplementary material:**

The online version of this article (doi:10.1186/s12888-016-0778-9) contains supplementary material, which is available to authorized users.

## Background

The negative symptoms of schizophrenia, characterized by the absence or loss of certain behaviors such as flat affect, account for most of the poor functional outcomes in patients with schizophrenia [1]. This core feature of schizophrenia includes both primary persisting negative symptoms and secondary negative symptoms that are related to extrapyramidal symptoms (EPS), depressive and/or psychotic symptoms. Therapeutic interventions in this disease with existing antipsychotics elicit partial beneficial effects on some of the key symptoms, however negative symptoms still persist in most of the patients [2]. As a consequence, many patients are left with negative symptoms even after their positive symptoms have been mitigated.

Additionally the majority of patients with schizophrenia do not fully respond to antipsychotics, and it has been reported that approximately 70 % of patients treated with antipsychotics do not achieve symptomatic remission after 3 years treatment [3]. The remaining positive symptoms of schizophrenia significantly increase the risk of relapse and re-hospitalization [3], and therefore decrease QOL and functioning [4, 5]. This population of patients referred to as “sub-optimally” treated patients represents a majority of schizophrenic patients.

A strong unmet need for better treatments, including safe adjunctive treatments that can be given in combination with currently available antipsychotics would undoubtedly exists to improve therapeutic efficacy against the above-mentioned symptoms [6–8].

Bitopertin is a small molecule with a novel mode of action that was designed on a basis of the working hypothesis that the hypo-functioning of the glutamatergic receptors within the brain, particularly the N-methyl-D-aspartate (NMDA) receptors (NMDA-R) [9, 10], is implicated in the pathophysiology of schizophrenia. This working hypothesis is repeatedly supported by the variety of pharmacological activities elicited by phencyclidine, ketamine and/or other NMDA receptor antagonists, because these compounds consistently induce clinical symptoms that are reminiscent of schizophrenia. Either phencyclidine or ketamine administrations to experimental animals produces positive- or negative- symptom like behavioral changes and cognitive deteriorations in these animals [11]. As glycine is reported to be an obligatory co-agonist of glutamate at the NMDA-R complex, one possible pharmacological intervention is to enhance the functions of the NMDA-R within the brain by elevating extracellular levels of glycine in the local microenvironment adjacent to the synaptic NMDA-R. Glycine elevations can be achieved through the inhibition of the glycine transporter 1 (GlyT1), which is known to be responsible for glycine removal from the synaptic cleft [9, 12]. An endogenous glycine transporter inhibitor, sarcosine, has been shown to be of help as short-term treatment for acutely ill schizophrenia [13, 14], chronically stable schizophrenia [15], and major depression [16], but not for treatment-resistant schizophrenia [17]. Bitopertin, a selective novel inhibitor of the GlyT1 and an orally active small molecule, is known to dose-dependently increase the extracellular levels of glycine in the striatum of the rat brain as well as in the cerebrospinal fluid of both the rat and healthy volunteers [18, 19].

This randomized double-blind clinical study was undertaken to evaluate the safety and efficacy profile of the three doses of bitopertin (5, 10, and 20 mg/day) as administering 52-week adjunctive treatment to existing antipsychotics in Japanese patients with “negative” symptoms or “sub-optimally controlled” symptoms, respectively. No placebo arm was established to prevent patients from dropping out from the study and to evaluate the long-term safety profile as the main purpose. Aside from this study, six global phase III studies including placebo arms were completed recently and the results are going to be published.

## Methods

### Study design

The present multi-center, randomized, double-blind phase III study was conducted at 35 clinical sites in Japan between August, 2011 and December, 2013, in compliance with the principles of the “Declaration of Helsinki” and in adherence with the “Good Clinical Practice” (JapicCTI-No.: JapicCTI-111627). The study protocol received institutional review board approvals prior to the study initiations (Additional file 1: List of the institutional review boards), and written, informed consent was given by all the participants prior to the study enrollments. Within 30 days after the informed consent was obtained, screening tests were performed, the eligibility of patients for this study was confirmed, and the stability of the patient’s symptoms was evaluated. Finally, patients were randomly assigned (1:5:5) to receive 52-week treatments with respective 5, 10, or 20 mg of bitopertin as an adjunct therapy to existing antipsychotics, which was followed by a 4-week follow-up period after the last dose of bitopertin was administered. The film-coated tablets for oral administration were manufactured by F. Hoffmann-La Roche, Ltd. For random allocations, a minimization method was employed to minimize imbalance between the dose groups in each of the two symptom groups in the factors including the type of the primary antipsychotics (atypical or typical) and age (under 65 or not). Glycine is essential for the heme synthesis in the erythroid progenitors and reticulocytes [20] and is taken into these cells via GlyT1 [21], and bitopertin, a glycine reuptake inhibitor, is known to potentially reduce the blood hemoglobin levels. Thus, the results of several relevant laboratory tests were blinded during the study period to prevent potential unblinding.

### Study population

The present study enrolled Japanese outpatients suffering from schizophrenia aged 18 and older. They were assigned to either group, the “negative symptom group” (stable patients with persistent, predominant negative symptoms of schizophrenia despite the treatments with antipsychotics) or the “sub-optimally controlled symptom group” (stable patients with insufficiently improved symptoms of schizophrenia despite the treatments with antipsychotics), respectively.

To be enrolled, participants were required to be stable on one or two antipsychotics with the exception of clozapine, whose total combined dose levels should not exceed 6 mg of risperidone equivalents, and to have hemoglobin levels of 12 g/dL or above because bitopertin potentially reduces hemoglobin levels.

The key inclusion criteria in the “negative symptom group” were, (1) a total score of greater than or equal to 40 on the sum of the 14 Positive and Negative Syndrome Scale (PANSS) [22] items constituting negative symptom factor score (NSFS) defined by the sum of PANSS items N1, N2, N3, N4, N6, G7, and G16, and PANSS disorganized thought/cognition factor scores defined by the sum of PANSS items P2, N5, G5, G10, G11, G13, and G15 [23], (2) a score of less than or equal to 22 on the sum of the 8 items of PANSS positive symptom factor score (PSFS) defined by the sum of PANSS items P1, P3, P5, P6, N7, G1, G9, and G12 [23], and a score of four on two or fewer of the items P1, P3, P6, and G9, and none with a score of five or higher. Additionally, (3) a score of four or higher based on Clinical Global Impression (CGI)–Severity of illness (CGI-S) negative symptoms [24] was required. The key exclusion criteria in the “negative symptom group” were, (1) depressive symptoms defined by Calgary Depression Scale for Schizophrenia (CDSS) score of nine or higher, and (2) a score of three or higher based on Clinical Global Impression of Movement Severity (CGI-MS) in the parkinsonism of the Extrapyramidal Symptoms Rating Scale–Abbreviated (ESRS-A).

The key inclusion criteria in the “sub-optimally controlled symptom group” were, (1) a PANSS total score of greater than or equal to 70, (2) a score of four or higher on two or more of the items P1, P3, P6, and G9, and (3) a score of four or higher based on CGI-S positive symptoms [24]. The key exclusion criteria in the “sub-optimally controlled symptom group” were patients in remission defined per all scores of three or lower on the PANSS items P1, P2, P3, G5, G9, N1, N4, and N6 [25].

### Assessment measures

The study visits were scheduled at screening, baseline, 2, 4, 6, 8, 12, 16, 20, 24, 28, 32, 36, 40, 44, 48, and 52 weeks from baseline. Medication compliance was checked by investigators at every visit.

The primary objective of the present study was to assess the safety. Safety evaluations, therefore, included adverse events (AEs), Columbia Suicide Severity Rating Scale (C-SSRS) for the assessment of suicidal tendency, laboratory tests, vital signs, 12-lead electrocardiogram, ESRS-A for the assessment of EPS, and ophthalmological examinations.

In this study, clinical efficacy evaluations were carried out as a secondary purpose. Trained and certified raters were solely instructed to assess PANSS scores. Both PANSS NSFS and PANSS PSFS were thought to be the most suitable endpoints for evaluating each respective “negative” and “sub-optimally controlled” symptoms because these were used as primary endpoints in global bitopertin phase III studies being carried out. Efficacy parameters included CGI-S, CGI–Improvement (CGI-I) [24], and Personal and Social Performance (PSP) [26]. Negative symptoms assessed both in terms of severity, CGI-S, and improvement, CGI-I, were included for the evaluation of the “negative symptom group”. Similarly, positive symptoms assessed both CGI-S and CGI-I were included for the evaluation of the “sub-optimally controlled symptom group”.

### Statistical analysis

The safety population consisted of all the patients who received at least one dose of the study medication. All the efficacy analyses were based on the intent-to-treat (ITT) population that comprised all the randomized patients who received at least one dose of the study medication and who had at least one post-baseline efficacy assessment.

The demographic characteristics of patients enrolled in the present study, and all the safety and efficacy measurements were summarized using descriptive statistics. The efficacy analyses were performed in each two symptom groups. Statistical hypothesis testing was not conducted, because the primary purpose of the present study was to evaluate the safety profile of bitopertin. The data were analyzed using SAS (version 9.1, Cary, NC, USA).

A target sample size planned was 165 patients (5 mg, 15 patients; 10 mg, 75 patients; 20 mg, 75 patients). This sample size was not determined according to the power calculation, but was determined to meet the requirement of a Japanese guideline of the health authority for safety evaluation.

## Results

### Patient disposition

**Fig. 1:**
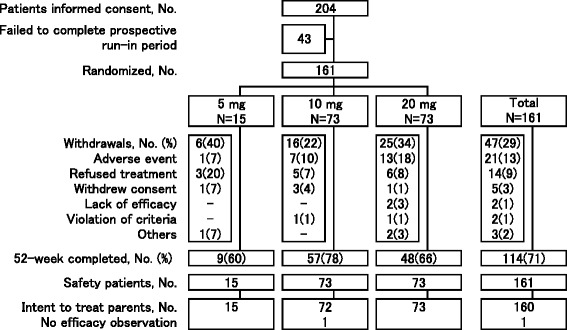
Patient flow diagram

Out of 204 patients who gave informed consents, 161 completed a prospective run-in period and were randomized to the three dosing arms (Fig. 1). Fifteen patients, 73 patients, and 73 patients were assigned to the 5, 10, and 20-mg groups, respectively. A total of 161 patients were thus treated with bitopertin. While 114 out of 161 patients (71 %) completed the 52-week treatment period, 47 patients (29 %) did not complete the 52-week treatment period.

The reasons for the withdrawals were “adverse event” in 21 patients (13 %), “refused treatment or did not cooperate” in 14 patients (9 %), “withdrew consent” in five patients (3 %), “insufficient therapeutic response” in two patients (1 %), “violation of inclusion and exclusion criteria” in two patients (1 %), and “others” in three patients (2 %), respectively. No apparent differences were observed among the dose groups in the proportions of patients withdrawn and their reasons.

### Patient baseline and demographic characteristics

One hundred five patients (65 %) were enrolled to the “negative symptom group”, and 56 patients (35 %) were to the “sub-optimally controlled symptom group”. The majority of the patients were on one of the following primary atypical antipsychotic drugs: risperidone, olanzapine, aripiprazole, or paliperidone (Table 1).

**Table 1:** Demographic characteristics and primary antipsychotic treatment

Characteristics	5 mg (*n* = 15)	10 mg (*n* = 73)	20 mg (*n* = 73)
Demographics			
Male, *n* (%)	12 (80)	51 (70)	47 (64)
Age in years, mean (SD)	41.8 (11.9)	39.9 (12.2)	41.8 (13.8)
BMI (kg/m^2^), mean (SD)	27.0 (4.9)	26.0 (4.2)	25.4 (4.9)
Schizophrenia type, *n* (%)			
Paranoid	11 (73)	43 (59)	38 (52)
Disorganized	1 (7)	11 (15)	10 (14)
Catatonic	-	2 (3)	-
Undifferentiated	-	10 (14)	7 (10)
Residual	3 (20)	7 (10)	18 (25)
Previous antipsychotics frequently used, *n* (%)			
Risperidone	4 (27)	8 (11)	12 (16)
Aripiprazole	2 (13)	6 (8)	13 (18)
Olanzapine	1 (7)	7 (10)	9 (12)
Primary antipsychotic treatment			
Type of Antipsychotics, *n* (%)			
Atypical	14 (93)	67 (92)	68 (93)
Typical	1 (7)	6 (8)	5 (7)
Route, *n* (%)			
P.O.	11 (73)	69 (95)	67 (92)
Infusion	4 (27)	4 (5)	6 (8)
Primary Antipsychotics, *n* (%)			
Risperidone	4 (27)	18 (25)	17 (23)
Olanzapine	5 (33)	19 (26)	15 (21)
Aripiprazole	2 (13)	16 (22)	20 (27)
Paliperidone	1 (7)	4 (5)	9 (12)
Others	3 (20)	16 (22)	12 (16)

*Abbreviations*: *SD* standard deviation

The ratios of males to females were 12/3, 51/22, and 47/26 in the 5, 10, and 20-mg groups, respectively. In these three groups, the means of age (and ranges) were 41.8 (22–64), 39.9 (18–67), and 41.8 (18–70) years, respectively. Thus, no apparent imbalances among the three dose groups were observed.

### Efficacy outcome

The results of the following efficacy measures that were evaluated at the last observation (at Week 52 or at the timing of a discontinuation of treatment) are shown in Table 2, including the mean changes from baseline in PANSS factor scores [23], CGI-S, PSP, proportions of responders (a 20 % or greater improvement) in PANSS NSFS/PANSS PSFS, and proportions of responders (“very much improved” or “much improved”) in CGI-I.

**Table 2:** Results of efficacy measures at last observation

	Negative symptom			Sub-optimally controlled symptom		
	5 mg (*n* = 10)	10 mg (*n* = 47)	20 mg (*n* = 48)	5 mg (*n* = 5)	10 mg (*n* = 25)	20 mg (*n* = 25)
PANSS, mean (SD)						
Total score						
Baseline	78.8 (9.9)	79.7 (10.3)	80.5 (7.4)	87.2 (10.4)	88.4 (12.2)	91.0 (13.6)
Change	−7.7 (5.7)	−11.0 (12.5)	−10.3 (15.9)	−9.4 (12.7)	−11.0 (14.0)	−7.5 (14.6)
Negative symptom factor score						
Baseline	25.9 (4.5)	26.7 (4.7)	26.8 (4.4)	22.2 (6.2)	22.6 (5.7)	24.2 (6.4)
Change	−4.8 (4.1)	−4.9 (4.7)	−4.9 (6.0)	−1.6 (3.7)	−3.7 (4.9)	−2.5 (4.8)
Positive symptom factor score						
Baseline	16.9 (3.3)	17.3 (3.5)	18.0 (3.2)	26.2 (6.0)	26.4 (3.8)	26.5 (5.1)
Change	−0.9 (0.9)	−1.7 (2.8)	−1.4 (3.8)	−4.2 (5.0)	−3.9 (4.5)	−2.1 (5.9)
Disorganized thought/cognition factor score						
Baseline	19.3 (2.5)	19.3 (4.3)	19.2 (3.0)	18.2 (2.9)	19.2 (3.7)	20.8 (4.8)
Change	−1.7 (1.9)	−2.5 (3.2)	−2.3 (3.6)	−1.8 (1.8)	−1.8 (3.0)	−1.4 (3.0)
Uncontrolled hostility/excitement factor score						
Baseline	6.5 (2.0)	7.1 (2.4)	7.0 (2.3)	10.4 (4.4)	9.5 (3.2)	9.0 (2.5)
Change	0.5 (1.2)	−0.6 (1.6)	−0.5 (2.2)	−1.0 (1.4)	−0.9 (2.3)	−0.7 (2.0)
Anxiety/depression factor score						
Baseline	10.2 (2.4)	9.3 (2.6)	9.6 (2.8)	10.2 (2.5)	10.7 (2.7)	10.5 (2.8)
Change	−0.8 (1.2)	−1.3 (2.2)	−1.3 (2.3)	−0.8 (1.9)	−0.8 (2.1)	−0.9 (2.5)
Responder in each symptom^a^, *n* (%)	6 (60)	27 (57)	23 (49)	2 (40)	14 (56)	7 (28)
CGI, mean (SD)						
CGI-S of overall symptom						
Baseline	3.6 (0.5)	4.1 (0.6)	4.2 (0.6)	4.2 (0.4)	4.2 (0.5)	4.4 (0.6)
Change	−0.1 (0.3)	−0.6 (0.9)	−0.6 (1.2)	−1.0 (1.0)	−0.8 (1.0)	−0.6 (1.0)
CGI-S in each symptom						
Baseline	4.4 (0.7)	4.4 (0.6)	4.5 (0.7)	4.2 (0.4)	4.3 (0.6)	4.3 (0.6)
Change	−0.8 (1.0)	−0.8 (1.0)	−0.9 (1.2)	−0.8 (1.1)	−0.9 (1.2)	−0.6 (1.2)
CGI-I of overall symptom^b^, *n* (%)	0 (0)	8 (17)	12 (26)	1 (20)	7 (28)	3 (12)
CGI-I in each symptom^b^, *n* (%)	2 (20)	9 (19)	12 (26)	1 (20)	6 (24)	4 (16)
PSP total score, mean (SD)						
Baseline	54.0 (16.2)	50.0 (14.2)	46.5 (16.0)	45.4 (18.8)	48.8 (15.6)	46.1 (12.5)
Change	7.2 (7.3)	7.3 (11.1)	7.0 (13.8)	10.6 (14.4)	5.0 (7.4)	6.8 (11.6)

*Abbreviations*: *CGI* clinical global impression, *CGI-I* CGI*-*improvement, *CGI-S* CGI*-*severity of illness, *PANSS* positive and negative syndrome scale, *PSP* personal and social performance, *SD* standard deviation

^a^20 % or greater improvement of PANSS negative symptom factor score in the negative symptom group or of PANSS positive symptom factor score in the sub-optimally controlled symptom group. The scores in each item were transformed from 1–7 to 0–6

^b^Responder of CGI-I is defined as a patient has a response of “very much improved” or “much improved”

### Negative symptom group

**Fig. 2:**
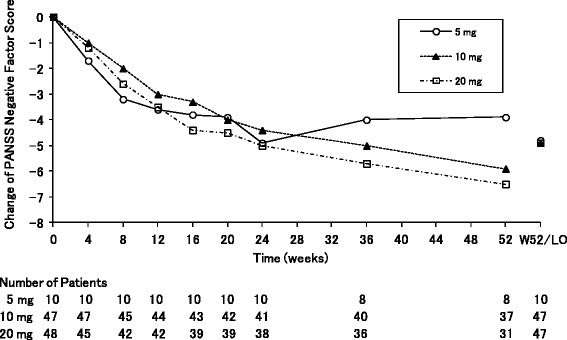
Mean changes in the PANSS negative symptom factor score in the negative symptom group. *Abbreviations*: *LO* last observation, *W52* Week 52

In the “negative symptom group”, the means of PANSS NSFS at baseline in each three dose groups were ranged from 25.9 to 26.8, and those of PANSS PSFS were ranged from 16.9 to 18.0, respectively. In all the three groups, PANSS NSFS gradually decreased from the first assessment point at Week 4 (Fig. 2). The mean changes from baseline of PANSS NSFS (standard deviation (SD), the numbers of patients) at the last observation were −4.8 (4.1, *n* = 10), −4.9 (4.7, *n* = 47), and −4.9 (6.0, *n* = 47) in the 5, 10, and 20-mg groups, respectively. In each three dose groups, the proportions of responders in the PANSS NSFS at the last observation were 60 %, 57 %, and 49 %, respectively. The mean changes (SD) of the PANSS total score at the last observation were −7.7 (5.7), −11.0 (12.5), and −10.3 (15.9) in each three dose groups, respectively.

The mean changes from baseline (SD) in CGI-S of the negative symptoms at the last observation were −0.8 (1.0), −0.8 (1.0), and −0.9 (1.2) in the 5, 10, and 20-mg groups, respectively. In each three dose groups, the proportions of responders in CGI-I of the negative symptoms at the last observation were 20 %, 19 %, and 26 %, respectively.

**Fig. 3:**
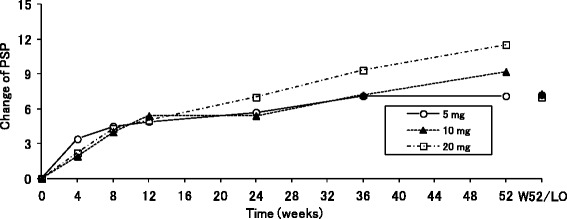
Mean changes in the personal and social performance total score in the negative symptom group. *Abbreviations*: *LO* last observation, *PSP* personal and social performance, *W52* Week 52

Furthermore, the PSP total score gradually increased from the first assessment point at Week 4 in all the three dose groups (Fig. 3). The mean changes (SD) of the PSP total score at the last observation were 7.2 (7.3), 7.3 (11.1), and 7.0 (13.8), in each three dose groups, respectively.

### Sub-optimally controlled symptom group

**Fig. 4:**
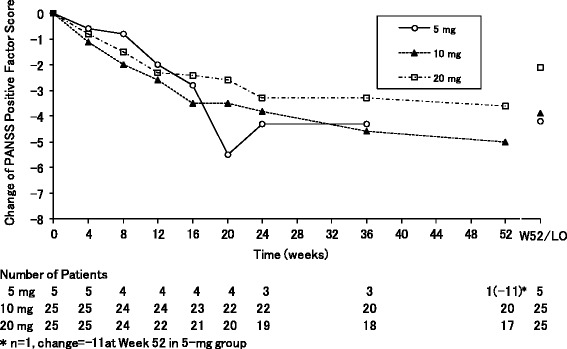
Mean changes in the PANSS positive symptom factor score in the sub-optimally controlled symptom group. *Abbreviations*: *LO* last observation, *W52* Week 52

In the “sub-optimally controlled symptom group”, the means of PANSS PSFS at baseline in each three dose groups were ranged from 26.2 to 26.5, and those of the PANSS NSFS were ranged from 22.2 to 24.2, respectively. In all the three dose groups, PANSS PSFS gradually decreased from the first assessment point at Week 4 (Fig. 4). The mean changes from baseline of PANSS PSFS (SD, number of patients) at the last observation were −4.2 (5.0, *n* = 5), −3.9 (4.5, *n* = 25), and −2.1 (5.9, *n* = 25) in the 5, 10, and 20-mg groups, respectively. In each three dose groups, the proportions of responders in the PANSS PSFS at the last observation were 40 %, 56 %, and 28 %, respectively. The mean changes (SD) of the PANSS total score at the last observation were −9.4 (12.7), −11.0 (14.0), and −7.5 (14.6) in each three dose groups, respectively.

The mean changes from baseline (SD) in CGI-S of the positive symptoms at the last observation were −0.8 (1.1), −0.9 (1.2), and −0.6 (1.2) in the 5, 10, and 20-mg groups, respectively. In each three dose groups, the proportions of responders in CGI-I of the positive symptoms at the last observation were 20 %, 24 %, and 16 %, respectively.

**Fig. 5:**
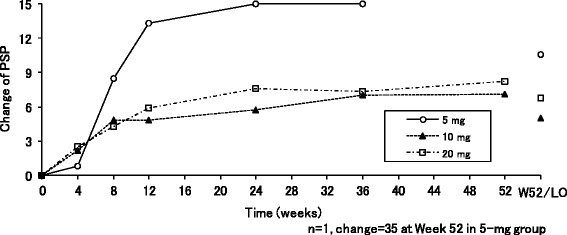
Mean changes in the personal and social performance scale score in the sub-optimally controlled symptom group. *Abbreviations*: *LO* last observation, *PSP* personal and social performance, *W52* Week 52

Furthermore, the PSP total score gradually increased from the first assessment point at Week 4 in all the three dose groups (Fig. 5). The mean changes (SD) of the PSP total score at the last observation were 10.6 (14.4), 5.0 (7.4), and 6.8 (11.6) in each three dose groups, respectively.

### Safety measures

In total, 411 AEs were reported in 142 out of 161 patients (88.2 %) (Table 3). AEs included nasopharyngitis (39.1 %), somnolence (16.8 %), worsening of schizophrenia (10.6 %), headache (7.5 %), insomnia associated with worsening schizophrenia (7.5 %), and other events (5.0 % or less), respectively. The incidences of AEs increased dose-dependently, and were 73.3 %, 87.7 %, 91.8 %, in the 5, 10, and 20-mg groups, respectively. Regarding the AEs with the incidence of ≥5.0 %, both somnolence and insomnia associated with worsening schizophrenia took place in a dose related manner, i.e., in the 5, 10, and 20-mg groups, the respective incidences of somnolence were 0 %, 12.3 %, 24.7 %, and those of insomnia were 0 %, 4.1 %, 12.3 %, respectively.

**Table 3:** Common AEs and ADRs

	5 mg (*n* = 15)	10 mg (*n* = 73)	20 mg (*n* = 73)	Total (*n* = 161)
Patients with at least 1 AE, *n* (%)	11 (73.3)	64 (87.7)	67 (91.8)	142 (88.2)
Total number of AEs, *n*	23	165	223	411
AEs with an incidence of more than 5 %				
Nasopharyngitis	3 (20.0)	31 (42.5)	29 (39.7)	63 (39.1)
Somnolence	-	9 (12.3)	18 (24.7)	27 (16.8)
Worsening of schizophrenia^a^	2 (13.3)	4 (5.5)	11 (15.1)	17 (10.6)
Headache	-	5 (6.8)	7 (9.6)	12 (7.5)
Insomnia associated with worsening schizophrenia^b^	-	3 (4.1)	9 (12.3)	12 (7.5)
Patients with at least 1 ADR, *n* (%)	2 (13.3)	35 (47.9)	41 (56.2)	78 (48.4)
Total number of ADRs, *n*	2	56	81	139
ADRs with an incidence of more than 2 %				
Somnolence	-	8 (11.0)	18 (24.7)	26 (16.1)
Worsening of schizophrenia^a^	1 (6.7)	4 (5.5)	7 (9.6)	12 (7.5)
Insomnia associated with worsening schizophrenia^b^		2 (2.7)	4 (5.5)	6 (3.7)
Parkinsonism	1 (6.7)	4 (5.5)	-	5 (3.1)
Headache	-	1 (1.4)	3 (4.1)	4 (2.5)
Akathisia	-	1 (1.4)	3 (4.1)	4 (2.5)

*Abbreviations*: *ADR* adverse drug reaction, *AE* adverse event

^a^“Worsening of schizophrenia” was coded to “schizophrenia” by Medical Dictionary for Regulatory Activities (MedDRA) version 14.1 preferred term

^b^“Insomnia associated with worsening schizophrenia” was coded to “insomnia related to another mental condition” by MedDRA version 14.1 preferred term

In total, 139 adverse drug reactions (ADRs) were reported in 78 out of 161 patients (48.4 %) (Table 3). ADRs included somnolence (16.1 %), worsening of schizophrenia (7.5 %), insomnia associated with worsening schizophrenia (3.7 %), parkinsonism (3.1 %), and other events (2.5 % or less), respectively. The incidences of ADRs dose-dependently increased, and were 13.3 %, 47.9 %, 56.2 %, in the 5, 10, and 20-mg groups, respectively.

No apparent differences were observed in the incidences of severe AEs among the three dose groups. The incidences of severe AEs were 6.7 % (one patient), 2.7 % (two patients), 2.7 % (two patients), in the 5, 10, and 20-mg groups, respectively. The severity of the most AEs were mild or moderate.

Nine serious AEs occurred in eight out of 161 patients (5.0 %). In the serious AEs, the causal relationships to bitopertin were not ruled out in three cases of worsening of schizophrenia i.e., two cases and one case in the respective 10 and 20-mg groups, and one case of neuroleptic malignant syndrome in the 10-mg group. Neuroleptic malignant syndrome occurred 4 weeks later after a 3-month treatment with bitopertin, and the patient started to take olanzapine prior to the event during the follow-up period.

The proportion of patients with AEs leading to drug discontinuations was as low as 13.0 %, and the proportion increased dose-dependently (6.7 %, 9.6 %, 17.8 %, in the 5, 10, and 20-mg groups, respectively). There were no AEs indicating drug dependency or withdrawal symptoms. Furthermore, the incidences of AEs were similar in both the “negative symptom group” and “sub-optimally controlled symptom group”, i.e., 88.6 % (93/105 patients; 249 events) and 87.5 % (49/56 patients; 162 events), respectively.

**Fig. 6:**
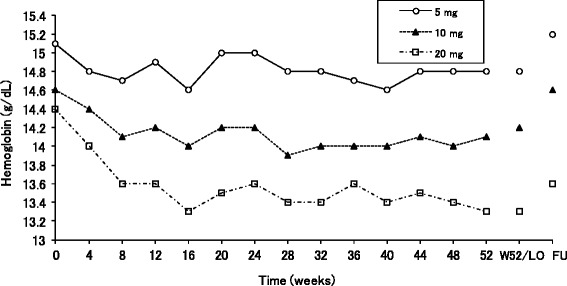
Mean hemoglobin levels. *Abbreviations*: *FU* after a 4-week follow up period, *LO* last observation, *W52* Week 52

The mean blood hemoglobin levels decreased gradually in a dose-related manner from baseline, and the values remained stable throughout the treatment periods examined, i.e., after Week 8 up to Week 52 in all the three dose groups (Fig. 6). These mean changes were within the normal range. After a 4-week follow-up period, the values returned to baseline in both the 5 and 10-mg groups, while the values did not return to baseline in the 20-mg group. In two patients, however, the blood hemoglobin levels met the withdrawal criteria during the study (decreases of 25 % and more from baseline), while the levels increased after drug withdrawals. The levels in one patient were 17.2, 12.7, and 14.5 g/dL at baseline and Week 32 (drug withdrawal), and after a 4-week follow-up period, respectively, and those in the other patient were 16.3, 11.8, and 14.4 g/dL at baseline and Week 16 (drug withdrawal), and after a 4-week follow-up period, respectively. In the two patients, bleeding was not reported. No other patients met the other withdrawal criteria related to the blood hemoglobin levels of under 10 g/dL.

Finally, no clinically relevant changes in vital signs, laboratory tests, 12-lead electrocardiogram, and ESRS-A were observed.

## Discussion

The hypo-functioning of NMDA-R within the brain has attracted great attention from neuroscientists as well as psychiatrists as one of the possible pathogenetic mechanisms underlying schizophrenia [9, 10]. Bitopertin is a glycine reuptake inhibitor that is expected to eventually activate neuronal NMDA-R by increasing the concentrations of glycine in the synaptic cleft, since glycine is an obligatory co-agonist of glutamate at the site of NMDA-R complex within the brain. The present phase III, multi-center, randomized, double-blind study was undertaken primarily to investigate the safety profile of bitopertin, and secondarily to examine whether or not bitopertin alleviates symptoms in patients who exhibit two distinct clinical symptoms of schizophrenia; the “negative symptom” and the “sub-optimally controlled symptom”. Bitopertin was administered at doses of 5, 10, and 20 mg/day as an adjunctive treatment to existing antipsychotics for 52 weeks.

The present study demonstrated that long-term treatment with bitopertin for successive 52 weeks was safe and well tolerated as far as the three dose levels of 5, 10, and 20 mg/day of bitopertin were concerned. The overall safety profile of bitopertin was similar to those reported in the two global phase II studies previously conducted; one was performed as an 8-week adjunctive therapy in patients with negative symptoms [27], and the other was performed as a 4-week monotherapy in patients with acute exacerbation of schizophrenia [28]. In the present study, neither dose-dependent increases in the incidences nor those in the severity of the AEs viewed as clinically relevant, e.g., EPS, were observed even in the presence of existing antipsychotics. In other words, the concomitant administrations of bitopertin with existing antipsychotics did not aggravate the safety profiles of the antipsychotic drugs.

Regarding the clinical efficacy outcomes, in both of the two respective patient population with “negative symptoms” and “sub-optimally controlled symptoms”, all the endpoints improved throughout the treatment period, compared with the baseline. No clear-cut dose-related responses among the three dose groups, however, were observed.

As one of the features in the present study, most of the patients enrolled completed 52-week treatment with bitopertin (71 %). In a meta-analysis of maintenance treatment with antipsychotics, the proportions of patients completed study periods were 70 % in the antipsychotics group, and the mean study duration weighted by sample sizes of individual trials was 9 months [29]. Hence, the proportion of patients completed in the present study is comparable to those of previous trials. This finding indicates that the safety profile following long-term treatment with bitopertin is favorable where existing antipsychotics were concomitantly administered. The high proportion together with no apparent imbalance of the proportion among the three dose groups enabled evaluation of the safety profile following long-term treatment with bitopertin. In this regard, of particular interest was to test whether or not any detrimental influences of bitopertin on the hemoglobin levels would be seen after long-term treatment, because schizophrenia requires lifelong treatment. Glycine is essential for the heme synthesis in the erythroid progenitors and reticulocytes [20] and is taken into these cells via GlyT1 [21]. Thus, it seems plausible that chronic treatments with bitopertin, a glycine reuptake inhibitor, may cause decreases in hemoglobin levels. In the previous global phase II study with 8-week bitopertin treatment at doses ranging between 10 and 60 mg/day, dose-dependent decreases in the hemoglobin levels were also observed [27]. The present study revealed that the decreased levels of hemoglobin following long-term treatment with bitopertin remained within the normal range, and increased towards baseline after drug withdrawals, suggesting that bitopertin is a reversible glycine reuptake inhibitor whose hemoglobin decreasing effects may be clinically tolerable even at the highest daily dose level of 20 mg investigated. Nonetheless, it is important to note that, since the exclusion criterion of hemoglobin levels was established less than 12 g/dL, drawing conclusions about any long-term effects of bitopertin in patients whose hemoglobin levels are below this level may be difficult. Thus, further clinical studies undoubtedly need to be performed to clarify this issue.

As shown in the present study, most of the AEs observed were mild or moderate in their severity, and few were evaluated as serious or severe AEs. Among the three different doses of bitopertin examined, the patients in the 20-mg group experienced more AEs than the patients in the other two dose groups, i.e., 5-mg and 10-mg groups. The most commonly observed AEs with a dose-dependency were somnolence and insomnia associated with worsening schizophrenia. A proportion of the patients with AEs leading to drug discontinuations was as low as 13.0 %; in two patients of the 20-mg group, the hemoglobin levels decreased and met the withdrawal criteria, decreases of 25 % and more from baseline. Altogether, it can be concluded that bitopertin is tolerable even after the long-term administration period of 52-week.

Regarding the clinical efficacy of bitopertin, since no placebo group was established in the present study, it seems hard to evaluate it. This is partly because definite placebo effects were repeatedly reported in the variety of clinical studies where compounds for the treatments of various types of psychiatric disorders were included [30]. In any event, the efficacy of bitopertin will have to be considered based on the outcomes of the present study in conjunction with those of six other global phase III studies being conducted in which placebo arms were included.

All the efficacy endpoints in the present study, i.e., PANSS, PSP, CGI-S, CGI-I scores at the last observation, apparently improved in all the three treatment groups for both two respective patient population with “negative symptoms” and “sub-optimally controlled symptoms”. While no clear-cut dose-responses were manifested among the three dose groups, the improvements became clear from the first assessment made at Week 4 following administrations of bitopertin. Among others, noteworthy is a finding that clinically meaningful improvements of the PSP scores over seven points, a clinically relevant effect in stable patients [31], were observed at the last observation for both of the two symptoms.

## Conclusions

Bitopertin was administered at doses of 5, 10, and 20 mg as an adjunctive treatment to existing antipsychotics for 52 weeks in respective two patient population of stable “negative symptoms” or “sub-optimally controlled symptoms”. No clinically relevant AEs, laboratory test abnormalities, 12-lead electrocardiogram abnormalities, and others were observed. It can be envisaged that bitopertin was generally safe and well-tolerated for the treatment of schizophrenia. All the efficacy endpoints, i.e., PANSS, PSP, CGI-S, and CGI-I scores, gradually improved in all the treatment groups for both of the two symptoms throughout the treatment period.

## Additional file

Additional file 1: List of the institutional review boards. (PDF 32 kb)

## Abbreviations

ADRs: adverse drug reactions
AEs: adverse events
CGI: clinical global impression
CGI-I: CGI-improvement
CGI-S: CGI-severity of illness
EPS: extrapyramidal symptoms
ESRS-A: extrapyramidal symptoms rating scale-abbreviated
GlyT1: glycine transporter 1
NMDA: N-methyl-D-aspartate
NMDA-R: NMDA receptor
NSFS: negative symptom factor score
PANSS: positive and negative syndrome scale
PSFS: positive symptom factor score
PSP: personal and social performance
SD: standard deviation
