# Supplementary material for: A double-blind randomized study assessing safety and efficacy following one-year adjunctive treatment with bitopertin, a glycine reuptake inhibitor, in Japanese patients with schizophrenia
Source: BMC Psychiatry. 2016 Mar 15;16:66. doi: 10.1186/s12888-016-0778-9 (PMC4791769; doi:10.1186/s12888-016-0778-9)
Supplement: Additional file 1: — List of the institutional review boards. (PDF 32 kb) [file 12888_2016_778_MOESM1_ESM.pdf]

List of the institutional review boards

Hino Hospital
Hinaga General Center for Mental Care
Ainohanazono Hospital
Tanaka Hospital
Uenohara Hospital
Kuranari Psychiatric Clinic
Yatsushiro Kosei Hospital
Sapporo Hanazono   Hospital
Osaka Psychiatric Medical Center
Okayama Psychiatric Medical Center
Uesugi Clinic
Nagasaki University Hospital
Mental Support Soyokaze Hospital
Hannan Hospital
Shinabuyama Hospital
Kurayoshi Hospital
Tokushima university Hospital
Kyushu University Hospital
Fujita Kokoro Care Center
Nagasaki Medical Center of Psychiatry
Yokohama City University Hospital
Kusatsu Hospital
Heisei Hospital
Kyowa Hospital
Tomita Hospital
Kamo Seishin Medical Center
Sawa Hospital
Hiroshima First Hospital
Azumi General Hospital
Tosa Hospital
Yamamoto Hospital
Mental Clinic Minami
Hokuto Clinic Hospital
Kakamigahara Hospital
Kokorono Clinic Narimasu
